# Rotating Squares Look Like Pincushions

**DOI:** 10.1177/2041669516664741

**Published:** 2016-09-05

**Authors:** Stuart Anstis, Sae Kaneko

**Affiliations:** Department of Psychology, University of California, San Diego, USA; Research Institute of Electrical Communication, Tohoku University, Japan; Department of Psychology, University of California, San Diego, USA

**Keywords:** motion perception, illusion, shapes or objects

## Abstract

Rotating squares appeared to be distorted into pincushions with concave sides. These illusory shape changes were caused by a perceived compression along the curved path of motion.

The outline of a square that rotates about its own center at 0.5 to 1 rev/s, appears to be distorted into a pincushion with pointy corners and concave sides. This is not simply a display artefact, since a square printed on paper shows pincushioning when spun on a turntable.

We can define the distortion ratio of a pincushion as a percentage decrement—the ratio of its pinched width to the width of an undistorted square (Figure 1).

We noticed that nested concentric squares give a strong effect as compared with a single square (online Movie 1). The corners appear to stick out too far, and the straight sides of the square look curved inwards. We also noticed that the amount of apparent pincushion distortion increases with rotation rate (up to about 1.5 rev/s, when computer aliasing breaks up the pattern).

We measured the pincushion distortion by a nulling method. We noticed that the extent of “pincushioning” increased with angular velocity. So, we manipulated the objective shape of the “squares” to null the perceptual pincushioning, collecting data independently at various set speeds. On each trial, the rotation rate was randomly selected from the range 0.2, 0.4, 0.5, 0.6, 0.8, and 1.0 rev/s. The observer struck designated keys to change the rotating shape along a continuum from pincushion to barrel, until satisfied that the shape looked square, neither pincushion- nor barrel-distorted. Striking the space key recorded the setting for later analysis offline and randomized the starting shape, automatically starting the next trial. Thus, observers set the physical barrel distortion to null the illusory pincushion distortion.

Results are shown in Figure 2 (3 Os × 10 settings). This plot reveals that increasing the rotation rate increased the perceptual pincushion distortion, with a 6% convexity nulling the pincushioning effect at 1.0 rev/s.

Ansbacher (1944) reported that a rotating arc looked apparently shorter along its direction of motion. This was confirmed by Stanley (1968); Anstis, Stürzel, and Spillmann (1999); and Geremek, Stürzel, da Pos, and Spillmann (2002). These authors showed that all arcs contract through equal angles at a given speed, no matter what their radii. However, whereas rotating arcs simply look shorter, our rotating squares actually appeared to change shape.

Figure 3 shows how contractions along a curved circular path can make straight lines look curved. The square in Figure 3(a) has its center at O, with its corners Q, S lying on the outer, dashed circle and the mid-side points P, R, T lying on the inner circle. The width of the square, POT, forms a straight line, thus an angle of 180°. Suppose that rapid rotation contracts the arcs of all circles, so that the radii close up like a lady’s fan until the angle POT shrinks to (say) 120°. The corners Q, S and the mid-side point P, R, T are constrained to remain on the outer and inner circles, respectively. This will bend the previously straight side of the square QRS into a concave curve. It would be a geometric impossibility for these perceptual distortions to happen simultaneously all “around the clock.” But many illusions are inconsistent with any physical reality. For instance, the Fraser spiral illusion (Fraser, 1908) is basically a physical circle made of twisted rope. The brain uncritically accepts local votes that falsely indicate that the circle is a spiral and makes no global check that might reveal the presence of a true circle. Here, in a similar way, the brain accepts the local pincushion distortions that would be inconsistent with any physical polygon. The geometrical simulation in Figure 3 shows how tangential compression of the square makes its corners peaky and its sides concave—just like a perceptual pincushion.

**Figure 1.**
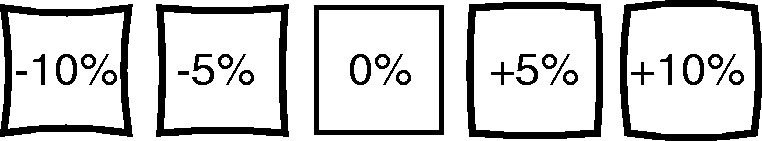
Pincushion distortion (concave sides) and barrel distortion (convex sides).

**Figure 2.**
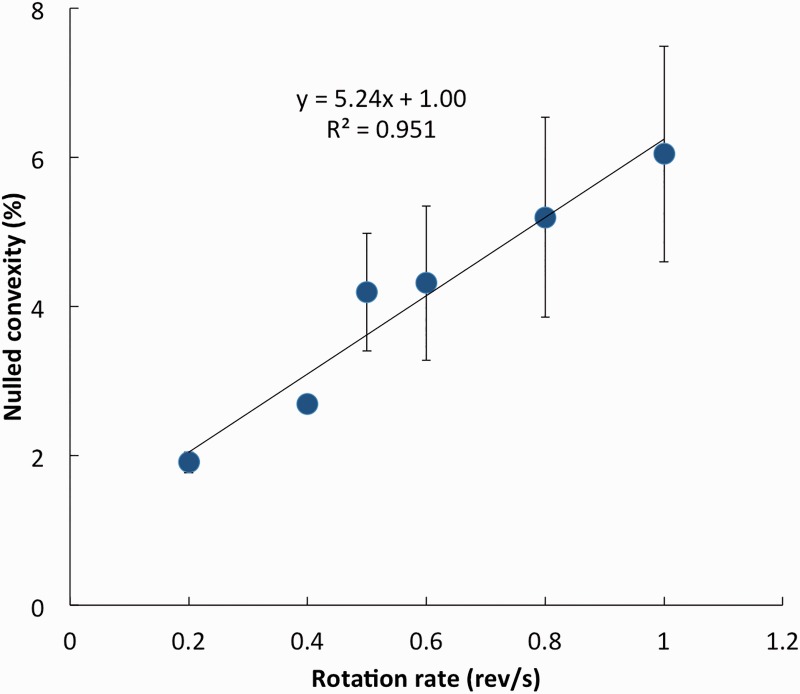
Pincushioning increased with rotation rates and was nulled by increasing amounts of barrel distortion. (Mean ± 1 SE of 3 Os.)

**Figure 3.**
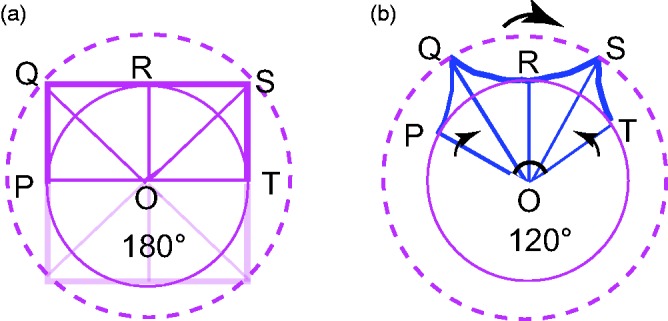
Plots of (a) an undistorted square. (b) Compression distorts the square into an apparent pincushion. Note that (b) shows upper half of square only.

## Supplementary Material

The online movie 1 is available at http://ipe.sagepub.com/supplemental

## References

[bibr1-2041669516664741] Ansbacher H. L. (1944) Distortion in the perception of real movement. Journal of Experimental Psychology 34: 1–23.

[bibr2-2041669516664741] Anstis S. Stürzel F. Spillmann L. (1999) Spatial distortions in rotating radial figures. Vision Research 39: 1455–1463. 10343814 10.1016/s0042-6989(98)00226-0

[bibr3-2041669516664741] Fraser J. (1908) A new illusion of visual direction. British Journal of Psychology 2: 307–320.

[bibr4-2041669516664741] Geremek A. Stürzel F. da Pos O. Spillmann L. (2002) Masking, persistence, and transfer in rotating arcs. Vision Research 42: 2509–2519. 12445845 10.1016/s0042-6989(02)00201-8

[bibr5-2041669516664741] Stanley G. (1968) Apparent length of a rotating arc-line as a function of speed of rotation. Acta Psychologica 28: 398–403. 5702208 10.1016/0001-6918(68)90028-0

